# Editorial: Neuroimaging study of post-stroke cerebral edema

**DOI:** 10.3389/fneur.2023.1167275

**Published:** 2023-03-07

**Authors:** Sheng Zhang, Chengcheng Zhu, Gabriel Broocks, Peiyu Huang, Christopher Blair

**Affiliations:** ^1^Department of Neurology, Center for Rehabilitation Medicine, People's Hospital of Hangzhou Medical College, Zhejiang Provincial People's Hospital, Hangzhou, China; ^2^Department of Radiology, University of Washington, Seattle, WA, United States; ^3^Department of Diagnostic and Interventional Neuroradiology, University Medical Center Hamburg-Eppendorf, Hamburg, Germany; ^4^Department of Radiology, School of Medicine, The Second Affiliated Hospital of Zhejiang University, Hangzhou, China; ^5^Sydney Brain Centre, Ingham Institute for Applied Medical Research Senior Lecturer SWS Clinical Campus, Faculty of Medicine and Health, UNSW Sydney, Sydney, NSW, Australia

**Keywords:** stroke, edema, imaging, identification, prediction

Stroke is a leading cause of death and long-term disability worldwide, affecting 10 million people each year (1, 2). Brain edema is one of the most harmful complications of stroke and can lead to further ischemic damage of brain tissue. Therefore, it is of great clinical importance to understand the causes of brain edema and identify biomarkers that can predict its occurrence. Imaging plays a crucial role in studying brain edema *in vivo*.

## Multi-modal CT scans

### Non-contrast CT scan

Since Broocks et al. developed quantitative net water uptake (NWU) based on non-contrast brain CT scans, several studies have been conducted on NWU in the field of brain edema (3). Two studies from Ningbo First People's Hospital (by Han et al. and Xu T. et al.) found that the occurrence of early edema in the ischemic core, as quantified by NWU, was time-dependent and correlated with the functional outcome of acute ischemic stroke patients. In addition, the NWU core/NWU penumbra ratio had good predictive power for the occurrence of symptomatic intracranial hemorrhage after endovascular therapy. Kumar et al. developed a method to automatically quantify the infarct area and NWU from brain non-contrast CT scans using deep learning, which may aid in exploring the factors influencing edema evolution in stroke patients in large-scale studies in the future.

In addition to NWU-related studies, Zhou et al. compiled a clinical-radiomics nomogram to characterize the relationship between brain edema and prognosis in patients with basal ganglia hemorrhage. They found that the radiomics features of peripheral hemorrhagic edema on non-contrast CT scans in patients with basal ganglia hemorrhage predicted patient outcomes. Application of this clinical-radiomic nomogram may therefore assist with individualizing clinical treatment decisions for these patients.

### CT perfusion (CTP) and CTP-derived 4-dimensional CT angiography (4D-CTA)

Shao et al. found that in patients who underwent successful endovascular therapy, a large mismatch ratio at baseline was a protective factor for rapidly progressive brain edema. Konduri et al. observed that collateral volume was the key factor affecting midline shift. Using CTP-reconstructed 4D-CTA, Chen et al. showed that an ipsilateral transverse sinus filling defect was related to the progression of brain edema and poor prognosis after stroke. Li et al. found that the volume of bloody fluids located between the temporal muscle and the targeted cerebral cortex, as calculated using 3D slicer software on non-contrast CT or CTA performed within 3 days post-surgery, affected the establishment of indirect collaterals in patients with moyamoya disease undergoing surgical bypass.

## Contrast-enhanced brain MRI

Intraventricular injection of a contrast agent and observation of cerebrospinal fluid metabolism by enhanced MRI is a recently validated method for evaluating glymphatic transport function in recent years. Zhang et al. studied glymphatic function using enhanced MRI (11.7T) by injecting gadolinium benzoate (BOPTA Gd) into the cistern magna of mice. They showed that the decrease in glymphatic clearance after stroke was related to cytotoxic edema caused by middle cerebral artery occlusion (MCAO), and that, the cytotoxic edema was related to the depolarization of aquaporin-4 in both the parenchyma PVSs and periventricular zone.

## Hemodynamics imaging

Pan et al. found that the affected-to-contralateral ratio of systolic cerebral blood flow velocity (CBFV) and mean CBFV were both independently associated with severe brain edema. They concluded that the affected-to-contralateral ratio of CBFV after endovascular treatment (EVT) may be a promising predictor of brain edema severity in patients who received EVT. Xu L. et al. found that cerebral vascular hemodynamic characteristics based on digital subtraction angiography (DSA), including flow direction and hemodynamic sources, can predict predict post-opertaive brain edema in surgically-managed Moyamoya disease.

## Texture analysis and machine learning

Shan et al. performed an in-depth analysis of CT images based on texture analysis, demonstrating its great potential in the non-invasive assessment of intracranial hypertension. Liu et al. established a machine learning model based on CT radiommic features and verified that it can predict the pressure amplitude correlation index (RAP) level of patients with severe traumatic brain injury, also providing a non-invasive method for intracranial pressure monitoring.

## Other research

Making ingenious use of the well-established clinical observation that pupil size reflects midbrain compression and midline shift in patients with ischemic stroke, Kim et al. used automatic infrared pupillary instruments to perform quantitative pupil observations on NICU patients and developed the neuropupillary index (NPi). Their results showed that the NPi had the potential to correlate with the degree of midline/pineal shift, suggesting that this technology could be used to monitor the occurrence and progression of brain edema in a high-dependency setting.

## Future directions

Brain edema after stroke is an active area of research that has seen increasing attention in recent years. In this Research Topic, 15 articles were published, including 1 review and 14 original studies. Based on these studies, we aim to further investigate the mechanism of brain edema and the clinical applications of imaging markers in the future, with the following core objectives. Firstly, most current studies are cross-sectional and have limited sample sizes, so future longitudinal studies and randomized controlled trials are needed to establish imaging markers that can predict brain edema in stroke patients. Secondly, as well as having a short scan time multi-model CT has the advantage of providing ample information about brain tissue and vessels, making it useful for evaluating brain edema in the early stages of stroke. However, most of these markers have not been prospectively verified. The same issue applies to the study of hemodynamic imaging using DSA and transcranial Doppler (TCD). Despite the great potential of hemodynamics analysis based on DSA and TCD, additional hemodynamic-related markers need to be developed and validated in future studies. Machine learning has great potential for accelerating imaging analysis, reconstructing images, and automating image analysis, but it is likely to have the greatest utility when applied to imaging markers that have been prospectively verified or have high consistency in manual measurement. Finally, research into the relationship between post-stroke glymphatic clearance and brain edema has become popular since 2020 (4). In this Research Topic, one study showed that post-stroke brain edema is related to the decline of glymphatic clearance function. Currently, there are very few imaging markers (especially non-invasive imaging) that can directly measure glymphatic function. Technical advancement in this field may provide tools for understanding the association between glymphatic function and brain edema. In particular, adjuvant treatment strategies for ischemic stroke beyond reperfusion are of significant clinical interest. To fully realize the potential of neuroprotectants and anti-edema drugs currently being tested in clinical trials neuroimaging tools, like those described here, are likely to be instrumental in assessing treatment effects and guiding optimal use in clinical practice.

## Author contributions

SZ was responsible for writing the editorial. CZ, GB, PH, and CB were responsible for proofreading, editing, and reviewing. All authors contributed to the article and approved the submitted version.
